# Screening and Prognostic Value of Methylated Septin9 and its Association With Clinicopathological and Molecular Characteristics in Colorectal Cancer

**DOI:** 10.3389/fmolb.2021.568818

**Published:** 2021-05-20

**Authors:** Jie Sun, Jinling Xu, Chao Sun, Minying Zheng, Yuwei Li, Siwei Zhu, Shiwu Zhang

**Affiliations:** ^1^ Department of Pathology, Tianjin Union Medical Center, Tianjin, China; ^2^ Graduate School, Tianjin University of Traditional Chinese Medicine, Tianjin, China; ^3^ Department of Colorectal Surgery, Tianjin Union Medical Center, Tianjin, China

**Keywords:** colorectal cancer, mSEPT9, prognosis, dMMR, genetic mutation

## Abstract

Screening of CRC continues to show poor compliance of endoscopy examination. The detection of mSEPT9 in peripheral blood is among the safe and simple early screening methods for CRC. The issue of how to elucidate whether detection of mSEPT9 in peripheral blood can effectively improve compliance of endoscopy and increase the early diagnosis rate of CRC and the relationship between levels of mSEPT9 in the peripheral blood and clinical stage, pathological classification, and expression of characteristic molecules in CRC remains unsolved. A total of 7759 individuals participated in the study that was performed using a questionnaire for screening of high-risk CRC. The endoscopic detection compliance of individuals with high-risk CRC who underwent the fecal occult blood test (FOBT) or mSEPT9 test was compared based on the results of the questionnaire. Additionally, correlation of mSEPT9 levels in the peripheral blood with clinicopathological features, mutation status of *TP53*, mismatch repair deficiency (dMMR), and *KRAS*/*NRAS*/*BRAF*/*PIK3CA* genotype was analyzed, and association of biomarkers with cancer-specific survival (CSS) and time to recurrence (TTR) was compared. We also detected levels of mSEPT9 in the peripheral blood of patients with CRC 7 days after surgery and compared the prognostic value of mSEPT9 with CEA. Results of our study showed that the mSEPT9 test could improve compliance of endoscopy and indicated a higher percentage of patients with positive mSEPT9 willing to undergo endoscopy detection than in those with positive FOBT. The specificity and sensitivity of mSEPT9 were better than that of FOBT for the detection of CRC. mSEPT9 was associated with the TNM stage, dMMR, and mutations in *TP53*, *BRAF,* and *PIK3CA*. A Ct value of mSEPT9 ≤ 37.5 was significantly related to poor CSS. mSEPT9 could affect association of dMMR and *BRAF* and *PIK3CA* mutations with CSS in a specific stage of CRC. The positive rate of mSEPT9 after surgery was found to correlate with poor TTR, and sensitivity was higher than CEA. The combination of mSEPT9 with CEA had a better prognostic value than that of mSEPT9 alone. The level of mSEPT9 was related to dMMR, mutations in *TP53*, *BRAF*, and *PIK3CA*, and was an effective biomarker for the prognosis of patients with CRC.

## Introduction

Colorectal cancer (CRC) is a common malignancy of the digestive tract. According to the GLOBOCAN data in 2018, with 1,800,000 cases, CRC ranked the third highest incidence rate among all malignancies detected worldwide. However, with about 880,000 deaths, CRC ranks the second in mortality rate for all malignant tumors globally (Bray et al., 2018). In recent years, screening methods such as fecal occult blood test (FOBT), endoscopy, and so on have been used for the early diagnosis and detection of CRC. However, low compliance with doctors’ advice remains a problem. Therefore, developing a simple and safe detection method with high specificity and sensitivity would significantly improve preliminary screening of CRCs and increase the compliance of patients.

Aberrant epigenetic changes, such as hypermethylation of CpG island, are the initiating events of colorectal tumorigenesis, and the gene hypermethylation status may serve as a useful biomarker for the screening of CRC (Rex et al., 2017; Danese et al., 2019). *Septin9* is a family of genes encoding conserved skeleton proteins with GTPase activity (Nagata et al., 2003). Detection of the *septin9* gene and protein in CRC confirmed their anticancer role in the occurrence and development of CRC. The hypermethylation of CpG island in the promoter of Septin9 can inhibit its normal gene expression and thus suppress the anticancer function (Toth et al., 2011). The detection of mSEPT9 in peripheral blood has been gradually adopted for the screening of CRC around the world (Sun et al., 2019). Moreover, our previous study has showed higher specificity and sensitivity of mSEPT9 detection in peripheral blood than that of FOBT (US Preventive Services Task Force et al., 2016).

Endoscopy is considered the best method of CRC screening, although it cannot effectively increase the diagnosis rate of CRC because of poor compliance by patients. In this study, we first focused on understanding whether mSEPT9 detection in peripheral blood can improve compliance to endoscopy and be used as an effective method for CRC screening. Further, although the treatment modalities for cancer, including chemotherapy, surgery, targeted therapy, and immunotherapy have significantly improved, the mortality rate of CRC remains high due to the complexity of its pathogenesis. CRC is caused by accumulation and interaction of multiple gene mutations and epigenetic changes. The specific mechanisms predominantly include chromosomal instability (CIN), microsatellite instability (MSI), and CpG island methylation phenotype (CIMP) (Simons et al., 2013; Overman et al., 2018). Mutations in KRAS, NRAS, BRAF, and PIK3CA mainly occur in CIN and are partially associated with CIMP. There is widely recognized molecular typing of CRC, although the correlation between various pathogeneses remains unclear, and the complex relationship among different pathogeneses of CRC increases the difficulty of individual therapy (Guinney et al., 2015; Alwers et al., 2019; Stintzing et al., 2019). Thus, it is of great clinical significance to explore effective molecular indicators for evaluation of molecular typing and prognosis of CRC. Cetuximab, a chimeric monoclonal antibody targeting the epidermal growth factor receptor (EGFR), has been used as targeted adjuvant chemotherapy of patients with CRC harboring wild-type KRAS, NRAS, BRAF, and PIK3CA genotypes. However, the influence of CIMP on the drug efficacy is still unclear. At present, studies about mSEPT9 mainly focus on screening, diagnosis, prognosis, and correlation analysis with histopathology, and Shen N et al. reported that the hypermethylation of SEPT9 associated with worse overall survival (OS) and disease-free survival (DFS) (Shen et al., 2019), but there were no systematic reports about the relationship between mSEPT9 with specific molecular characteristics of CRC and the prognosis value of adjuvant chemotherapy or recurrence. Our previous study showed that mSEPT9 is related to the TNM stage, Dukes stage, and dMMR status of CRC (Sun et al., 2019). Thus, second, we have focused on the correlation between mSEPT9 and clinicopathological features, including routine histopathology, status of *TP53* mutations, expression of mismatch repair (MMR) proteins, and *KRAS*, *NRAS*, *BRAF,* and *PIK3CA* genotypes. Third, the relation of these biomarkers with cancer-specific survival (CSS) and time to recurrence (TTR) in CRC patients treated with adjuvant chemotherapy was also studied. Study about mSEPT9 may provide newer molecular indicators for early diagnosis, treatment, prognosis evaluation, and recurrence monitoring of patients with CRC.

## Materials and Methods

### Study Population and Design

For screening of CRC, 7759 individuals in Tianjin aged >40 years participated in the survey. The screening was carried out by a questionnaire survey, followed by endoscopy, and detection of FOBT and mSEPT9 in the peripheral blood. The questionnaire included basic information of residents, history of intestinal diseases, family history of CRC, and so on. These points were in line with the following positive prompts for high-risk populations: 1) history of CRC or adenoma; 2) first-degree relatives with a history of CRC; 3) coexistence of two or more of the following history: chronic diarrhea, chronic constipation, mucus bloody stool, chronic appendicitis or appendectomy, unhealthy life, and chronic choledochocystitis or cholecystectomy; and 4) have a direct relative with familial adenomatous polyposis and hereditary nonpolyposis CRC (HNPCC). Further, people with a high risk of CRC were randomly divided into three groups, *viz.* high-risk, high-risk + FOBT, and high-risk + mSEPT9. Each participant was suggested to undergo an endoscopy examination, but the subjects could independently choose not to accept the endoscopy. The number of subjects accepting the endoscopy examination among the positive and negative subjects of FOBT and mSEPT9 was counted to compare the compliance of the endoscopy. Furthermore, the sensitivity and specificity between FOBT and mSEPT9 were evaluated.

**FIGURE 1:**
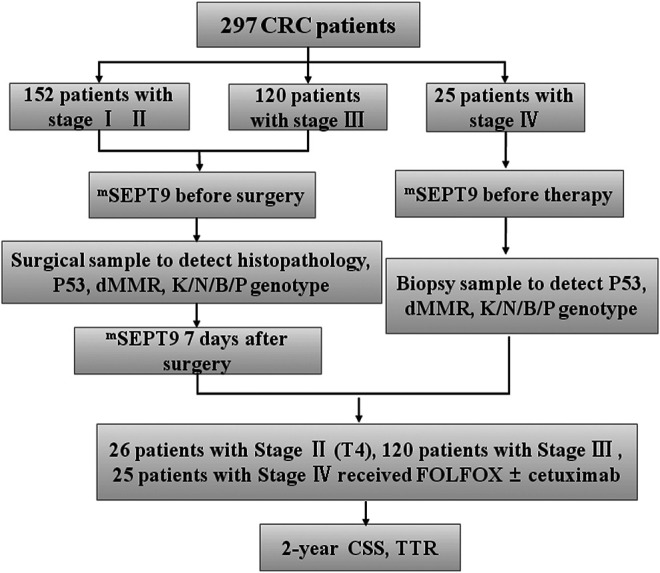
Flowchart of study design. Patients with CRC (*n* = 297), including 152 with stage I–Ⅱ, 120 with stage Ⅲ, and 25 with stage Ⅳ CRC. Peripheral mSEPT9 has been tested before surgery and treatment, followed by analyses of clinicopathological and molecular characteristics. The blood samples of patients with stages I–Ⅲ have been collected 7 days after surgery. Patients have been treated with the FOLFOX regimen with or without cetuximab, and the outcome of patients in CSS and TTR has been followed for 2 years. KNBP stands for *KRAS*/*NRAS*/*BRAF*/*PIK3CA*.

For evaluating the prognosis of mSEPT9 in a peripheral blood in training study, a database of patients who underwent the endoscopy examination and surgical treatment at the Tianjin Union Medical Center between January 2018 and April 2020 was retrospectively studied. The diagnosis and treatment programs were based on the guidelines of the National Comprehensive *Cancer* Network (NCCN) and Chinese Society of Clinical Oncology (CSCO) (Seal et al., 2014). Our study was performed according to the principles of the Declaration of Helsinki and was approved by the Research Ethics Committee of the Tianjin Union Medical Center, China. All the patients provided written informed consent for a peripheral blood test and use of their paraffin-embedded CRC tissues for genetic analyses. The specific study protocols are listed in Figure 1.

The 297 CRC patients who were diagnosed by endoscopy included 272 patients with stages I–Ⅲ and 25 patients with stage IV disease, whose blood samples were collected before radical operation and medical treatment, respectively. The tissue samples of 297 patients with stage I–IV CRC were also collected, and clinicopathological and molecular characteristics were analyzed. The blood samples of 272 patients with stage I–Ⅲ CRC were collected 7 days after surgery. Patients with radical resection (stages I–Ⅲ) were recruited to study the feasibility of assessing the levels of mSEPT9 DNA in plasma for recurrence evaluation and compared with those of carcinoembryonic antigen (CEA). Patients with wild-type *KRAS*, *NRAS*, *BRAF*, and *PIK3CA* genotypes were treated using the FOLFOX regimen [folinic acid (leucovorin calcium), fluorouracil, and oxaliplatin] and cetuximab, while those with mutated *KRAS*, *NRAS*, *BRAF*, and *PIK3CA* genotypes were treated with FOLFOX alone. The final diagnosis of recurrence was drawn based on the results of the endoscopy and postoperative pathological examination. Here, the diagnosis of recurrence included local recurrence and distant metastasis. The results of FOBT and CEA were directly collected from the clinical laboratory. The outcomes of all the patients in CSS and TTR were followed for 24 months. In a validation study, the clinicopathological data of an additional 226 patients with CRCs in the Tianjin Union Medical Center from December 1, 2018 to March 31, 2019 were collected to evaluate the prognostic value of mSEPT9. The outcome of all the patients in CSS and TTR was followed for 24 months.

### Sample Collection and Storage

The peripheral blood samples (10 ml) were collected in test tubes containing K_2_EDTA anticoagulant (BD Biosciences, NJ, United States). Plasma samples (3.5 ml) were collected upon centrifugation and stored under −20°C.

### Quantification of Methylated Septin9

An improved *SEPT9* gene methylation assay (Epigenomics AG for Epi proColon 2.0) was used for the screening of CRC in our study. DNA was extracted from plasma samples using the plasma processing kit (BioChain Science and Technology, Inc., Beijing). The DNA was then incubated with bisulfite, and methylated target sequences in the bisulfite-converted DNA template were amplified using real-time PCR. The methylation of *SEPT9* in plasma samples was measured using an ABI7500 fluorescent PCR instrument. For Ct values ≤41, the result was considered positive, whereas for Ct values ≥41, the result was considered negative.

### Fecal Occult Blood Test

An immune colloidal gold technique was utilized for the detection of fecal occult blood (FOB), and monoclonal antibodies were used to specifically target human hemoglobin (HB) in feces samples. The reaction line (T) on the cellulose nitrate membrane was coated with an anti-HB1 monoclonal antibody, and the control line (C) was coated with a sheep anti-mouse polyclonal antibody. When detected, the human hemoglobin in the sample could bind to the colloidal gold–antibody coated at the front of the reagent to form an immune complex. As the chromatographic complex moves along the membrane band, if it is a positive sample, it can agglutinate to form a color band on the reaction line (T) and the control line (C), respectively. If it is a negative sample, it will only form a color band on the control line (C). The lowest detectable level of hemoglobin was 0.2 μg/ml, ranging from 0.2 μg/ml to 2000 μg/ml. For sample extraction, a 10–50 mg sample was taken from six different parts of stool with a stool bar and mixed well in 0.5 ml buffer solution for detection.

### Immunohistochemical Staining

Sections with 4 μm thickness of the CRC tissue samples were subjected to immunohistochemical analysis to detect the expression of mismatch repair proteins MLH1, MSH2, MSH6, PMS2, and p53. The MMR proteins expressed in normal intestinal epithelial cells, lymphocytes, smooth muscle cells, and vascular endothelial cells were used as the internal control. For a single mismatch repair protein, when the inner control cell nucleus was positive and cancer cell was negative (no nuclear staining), the protein expression was absent. In general, the absence of one or more MMR proteins in the tumor cells can be interpreted as MMR deficient (dMMR), and it corresponds to microsatellite instability–high (MSI-H) status. Whereas no deficiency of MMR proteins in tumor cells can be interpreted as MMR proficient (pMMR) and corresponds to microsatellite instability–low (MSI-L) or microsatellite stability (MSS) status. The absence of any of the proteins was interpreted as dMMR (Luchini et al., 2019). Each result was confirmed by two experienced pathologists.

### Mutation Analysis of *KRAS*/*NRAS*/*BRAF*/*PIK3CA*

The DNA from paraffin-embedded samples was extracted using the Qiagen extraction kit (Cat No.: 56404, Germany), and its genotype was determined using PCR analysis. We analyzed 12 somatic hot spot mutations in exons 2, 3, and 4 of the human *KRAS* gene, 3 somatic hot spot mutations in exons 2 and 3 of the human *NRAS* gene, V600E hot spot mutation in exon 15 of the human *BRAF* gene, and 5 hot spot mutations in exons 9 and 20 of the human *PIK3CA* gene.

### Statistical Analyses

All statistical analyses were performed using the SPSS version 25.0. ANOVA and *t* tests were performed to compare the differences of mSEPT9 levels among different groups, and to analyze the association of Ct values of mSEPT9 with specific molecules of dMMR. The survival analysis was based on CSS and TTR. The CSS was defined as the time from CRC diagnosis to CRC-related death or end of follow-up. TTR was calculated as the period from surgery to either death from CRC, local recurrence, or distal metastasis. The influence of biomarkers on CSS and TTR was analyzed using the Kaplan–Meier curves and Wilcoxon test. Hazard ratios (HRs) and 95% confidence intervals (CIs) for CRC-related deaths were estimated using Cox regression analysis. Multivariable adjusted models included the predefined potential prognostic factors, such as age at diagnosis, sex, tumor localization, and TNM stage. A two-sided *p* value < 0.05 was considered statistically significant.

## Results

### Detection of Methylated Septin9 Improves Compliance of Endoscopic Examination for Screening of Colorectal Cancer

Of the 7759 participants, 3578 were men with a median age of 55 years, while 4181 were women with a median age of 56 years. Based on the results of the questionnaire survey, there were 882 individuals with a high risk of CRC. Further, 105 subjects belonged to the high-risk group according to criterion (1), 449 belonged to criterion (2), 367 belonged to criterion (3), and 41 belonged to criterion (4) (some subjects meet two criteria). These criteria have been listed in the study population and design. These 882 individuals were randomly divided into the three groups, *viz.* high-risk, high-risk + FOBT, and high-risk + mSEPT9, and each group contained 294 subjects. The percentage of patients who volunteered individuals for endoscopy examination in the high-risk group, positive FOBT group, and positive mSEPT9 group were 23%, 42%, and 96%, respectively, which may be associated with the high sensitivity and specificity of the peripheral mSEPT9 test in the early screening of CRCs. The positive test result of mSEPT9 contributed to higher compliance of endoscopy examination than that of positive FOBT. The rate of endoscopy examination in the negative mSEPT9 group was 15% (Table 1). There were 55 patients with CRC confirmed by an endoscope biopsy. Of these, 31 were men with a median age of 64 years, and 24 were women with a median age of 67 years. Furthermore, 25 cases were detected in the colon and 30 in the rectum. The sensitivity and specificity of mSEPT9 were 73% and 98%, respectively, which were significantly higher than the respective 55% and 86% values for FOBT (Table 2). Thus, based on the results obtained, detection of peripheral blood mSEPT9 is an effective way to address the poor compliance of endoscopy examination, and the sensitivity and specificity of mSEPT9 detection are better than the clinical routine FOBT detection.

**TABLE 1:** Function of FOBT and mSEPT9 in endoscope compliance.

	No. of patients	Endoscopy examination	*p*
High-risk	294	68 (23%)	
High-risk + FOBT negative	242	39 (16%)	0.73^a^
High-risk + FOBT positive	52	22 (42%)	0.02^a^
High-risk + mSEPT9 negative	271	41 (15%)	0.81^a^
High-risk + mSEPT9 positive	23	21 (96%)	<0.001^a^, 0.01^b^

a *p* value for the comparison with the high-risk group.

b *p* value for the comparison between the mSEPT9 positive group and FOBT positive group.

**TABLE 2:** Sensitivity and specificity of mSEPT9 and FOBT detection in the screening of CRC for the high-risk population.

	Number of total CRC patients	Number of CRC patients with positive SEPT9 or FOBT result	Sensitivity (95%CI)	Number of total healthy participants	Number of healthy participants with negative SEPT9 or FOBT result	Specificity (95% CI)
mSEPT9	26	19	73% (62–81%)	264	260	98% (89–99%)
FOBT	29	16	55% (44–65%)	265	229	86% (81–90%)

### Correlation of Methylated Septin9 With Clinicopathological and Molecular Characteristics of Colorectal Cancer

**FIGURE 2:**
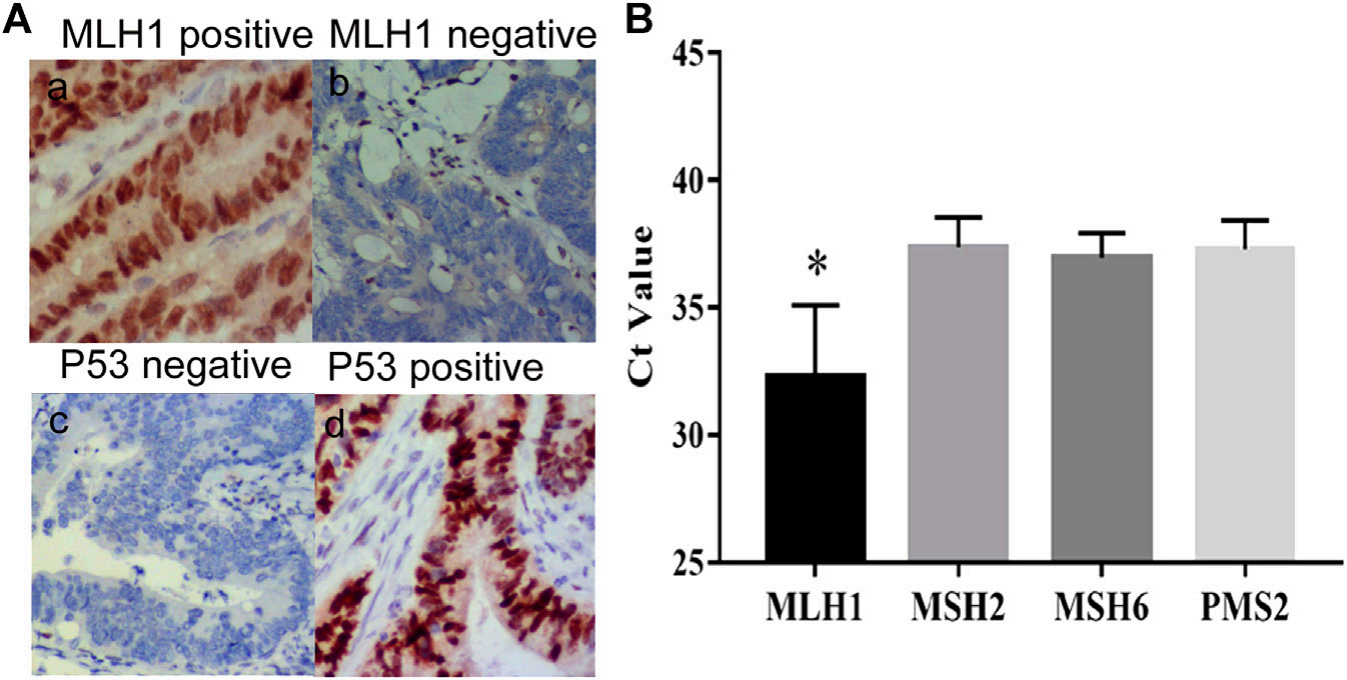
Expression of MLH1 and P53. **(A) (a)** Positive expression of MLH1 (IHC, magnification × 200). **(b)** Negative expression of MLH1 (IHC, magnification × 200). **(c)** Negative expression of P53 (IHC, magnification × 200). **(d)** Positive expression of P53 (IHC, magnification × 200). **(B)** The Ct values of mSEPT9 with specific molecules of dMMR. The Ct value of MLH1 is the highest among the four specific molecules of dMMR (^*^ *p* < 0.01).

The clinical characteristics of 297 CRC patients who underwent the procedure for detection of peripheral mSEPT9 are summarized in Table 3. The correlation analysis for the result of peripheral mSEPT9 with clinicopathological and molecular characteristics is summarized in Table 4. The mSEPT9 was not significantly associated with the location of CRC. With the increase in the TNM stage, the Ct values of mSEPT9 decreased, indicating an increased release of mSEPT9 protein by tumor cells into the peripheral blood. As per the TNM stage, the Ct value of mSEPT9 was highest in stage I, lowest in stage IV, and statistically insignificant in stages II and III. There were 59 cases of dMMR, which contributed to 19.9% of 297 cases with CRC. Moreover, the positive rate of mSEPT9 in stage II was higher than that in stage III, which may be due to occurrence of dMMR in stage II, and that the positive rate of mSEPT9 in dMMR was higher than that in pMMR. Furthermore, the negative expression of MLH-1 mainly occurred in positive mSEPT9 samples, while positive expression had a higher possibility of occurrence in negative mSEPT9 samples. These results further validated the association between mSEPT9 and dMMR (Figure 2A a and b). Additionally, the MLH-1 expression was highest in patients with positive mSEPT9 (Figure 2B). Of the 297 patients with CRC, 97 cases had the wild-type status of *TP53*, and 200 had mutant *TP53*. The Ct values of mSEPT9 in patients with TP53 missense mutation was less than that in those with wild-type TP53. Results of immunohistochemical staining showed that increased TP53 missense mutations occurred in the positive mSEPT9 group, indicating that hypermethylation of CpG island of *SEPT9* may promote *TP53* missense mutations in patients with CRC (Figure 2A c and d). The mutation rate in *KRAS*/*NRAS* was 49.2%, *BRAF* was 5.1%, and *PIK3CA* was 2% in all these 297 patients with CRC. In the study of the correlation between mSEPT9 and *KRAS*/*NRAS*/*BRAF*/*PIK3CA* mutation genes, mSEPT9 was related to *BRAF* and *PIK3CA* gene mutations but not to *KRAS* and *NRAS* mutations (Table 4), suggesting that mSEPT9 may promote the downstream molecule mutations in the EGFR signaling pathway.

**TABLE 3:** Clinical characteristics of 297 patients with CRC.

Variable	No. of patients
Age	
<40 years	12
40–50 years	34
51–60 years	72
61–70 years	112
>70 years	67
Gender	
Female	127
Male	170
History	
One first-degree relative with CRC	30
Intestinal adenoma or polyps	59
Chronic constipation	105
Chronic diarrhea	82
Inflammatory colon diseases	37
Mucus and bloody stool	64
Chronic appendicitis or appendectomy	11
Smoking	113
Drinking	202
Non	42

**TABLE 4:** Clinicopathological and molecular characteristics related to mSEPT9 in 297 cases of CRC.

Clinicopathological parameter	No. of patients	Positive rate (%)	Mean Ct value	*p*
**Localization**				
Colon	122 (41.1%)	90.2	37.4	
Rectosigmoid transition	35 (11.8%)	85.7	36.6	0.097
Rectum	140 (47.1%)	90.0	37.8	0.25
**Invasive depth**				
T1	19 (6.4%)	73.7	40.2	
T2	60 (20.2%)	88.3	38.7	0.034
T3	141 (47.5%)	86.5	37.9	0.049
T4	77 (25.9%)	100	35.2	0.016
**Lymph node metastasis**				
N0	152 (51.2%)	90.1	38.1	
N1	93 (31.3%)	89.2	36.6	0.11
N2	52 (17.5%)	88.5	37.4	0.082
**Distant metastasis**				
M0	272 (91.6%)	88.6	37.7	
M1	25 (8.4%)	100	34.9	0.008
**TNM stage**				
Stage I	24 (8.1%)	75	39.9	
Stage II	128 (43.1%)	93.0	37.7	0.035
Stage III	120 (40.4%)	86.7	37.3	0.077
Stage IV	25 (8.4%)	100	34.9	0.006
**Histopathological grade**				
High differentiation	104 (35.0%)	93.3	37.8	
High-middle/middle differentiation	59 (19.9%)	83.1	36.2	0.13
Middle-low/low	97 (32.7%)	90.7	38.0	0.078
Mucinous adenocarcinoma/signet-ring cell carcinoma	37 (12.5%)	86.5	37.1	0.19
**Gross tumor volume**				
0–10 cm^3^	155 (52.2%)	87.1	38.6	
>10 cm^3^	142 (47.8%)	92.3	36.3	0.012
**Morphology**				
Protruded type	120 (40.4%)	89.2	37.2	
Ulcerative type	126 (42.4%)	92.1	37.5	0.26
Mixed type	51 (17.2%)	84.3	38.1	0.098
***TP53* status**				
Wild-type	97 (32.7%)	85.6	39.2	
Mutated	200 (67.3%)	91.5	36.7	0.032
**MMR status**				
dMMR	59 (19.9%)	98.3	35.8	
pMMR	238 (80.1%)	87.4	37.9	0.040
***KRAS*/*NRAS* status**				
Wild-type	151 (50.8%)	90.1	37.7	
Mutated	146 (49.2%)	89.0	37.3	0.18
***BRAF* status**				
Wild-type	282 (94.9%)	89.0	37.6	
Mutated	15 (5.1%)	100	35.4	0.047
***PIK3CA* status**				
Wild-type	291 (98.0%)	89.3	37.6	
Mutated	6 (2.0%)	100	34.9	0.025

### Association Between Methylated Septin9 Levels, Specific Genotype Mutations, and Cancer-Specific Survival and Time to Recurrence

**FIGURE 3:**
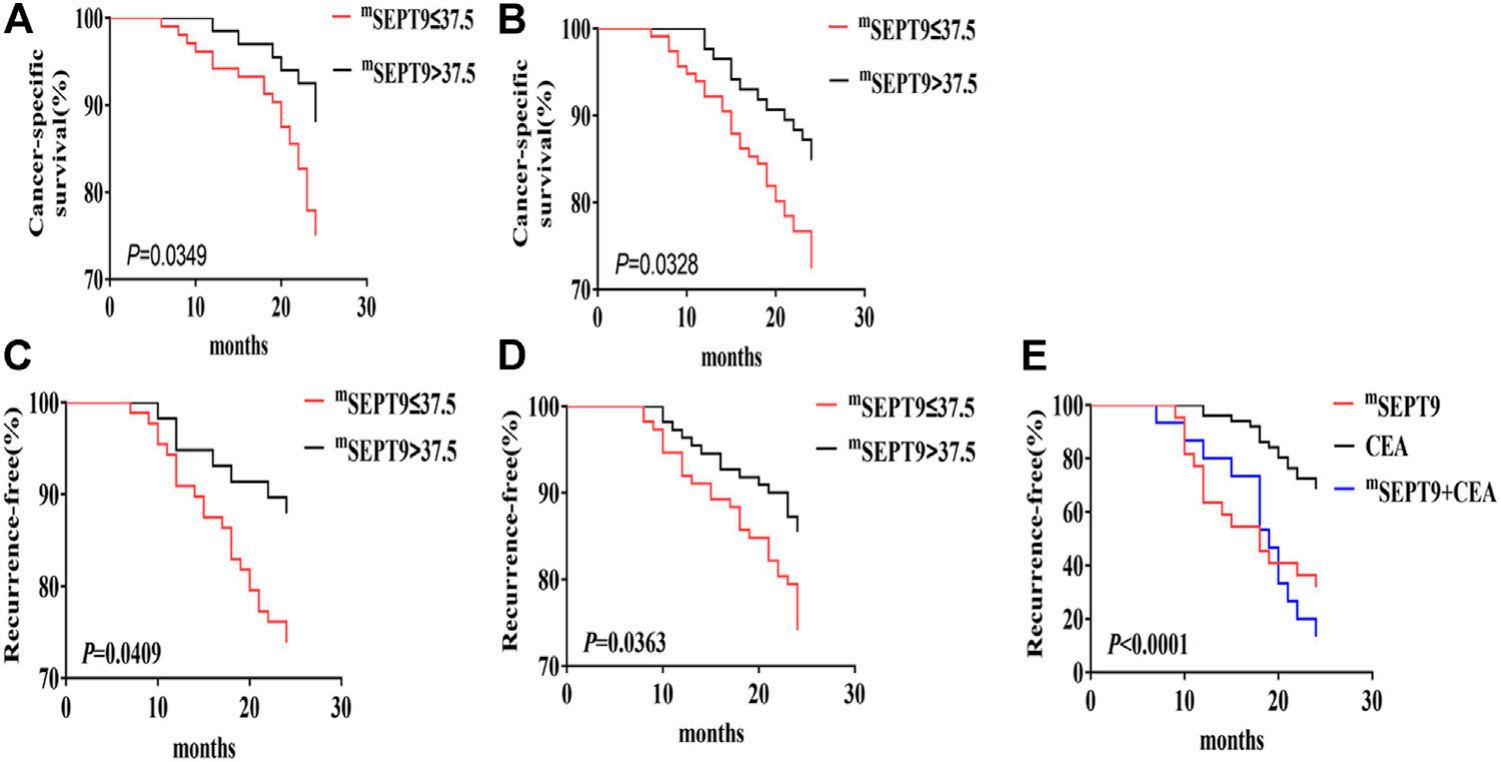
CSS and TTR analysis of mSEPT9. **(A)** Kaplan–Meier curves of CSS in the overall cohort based on the Ct value of mSEPT9 before surgery or treatment with adjuvant chemotherapy. **(B)** The validation study of Kaplan–Meier curves of CSS in the overall cohort based on the Ct value of mSEPT9 before surgery or treatment with adjuvant chemotherapy. **(C)** Kaplan–Meier curves of TTR in patients with stage I–Ⅲ CRC, based on the Ct value of mSEPT9 before surgery or treatment with adjuvant chemotherapy. **(D)** The validation study of Kaplan–Meier curves of TTR in patients with stage I–Ⅲ CRC, based on the Ct value of mSEPT9 before surgery or treatment with adjuvant chemotherapy. **(E)** Kaplan–Meier curves of TTR in patients with stages Ⅰ–Ⅲ, based on the Ct value of mSEPT9, CEA, and combination of mSEPT9 and CEA measured 7 days after surgery.

In patients with stage I–Ⅳ CRC, there was no significant difference in the CSS between positive mSEPT9 and negative mSEPT9 before treatment. However, CRC patients whose mSEPT9 Ct value ≤37.5 were associated with a shorter CSS than those with >37.5 Ct value (Table 5). Of the patients who received adjuvant chemotherapy, patients with mSEPT9 Ct value ≤37.5 had worse CSS than those with Ct value >37.5 (Figure 3A), which was consistent with the validation study result (Figure 3B). There was no significant difference for CSS between dMMR and pMMR in CRC patients, and the prognosis between dMMR and pMMR was not affected by mSEPT9 expression levels (Ct value ≤37.5). Patients with KRAS/NRAS-mutated tumors were associated with a shorter CSS than those with the wild-type status, although the Ct value of mSEPT9 had no significant aggravation of CSS on patients with KRAS/NRAS-mutated tumors. The patients with BRAF-mutated tumors had shorter CSS than those with BRAF wild-type tumors, and those with a Ct value of mSEPT9 ≤37.5 tumors harboring BRAF mutations had worse CSS than those with a Ct value of mSEPT9 >37.5 tumors harboring BRAF-mutated tumors. No significant difference was observed between patients with PIK3CA-mutated tumors and those with PIK3CA wild-type tumors, but the PIK3CA-mutated tumors with positive mSEPT9 (Ct value ≤37.5) correlated with worse CSS than those with Ct value of mSEPT9 > 37.5 tumors harboring PIK3CA mutation. Further, in the analysis of relationship of patients’ TTR and stage I–Ⅲ CRC (Table 5), the Ct value of mSEPT9 ≤37.5 was associated with shorter TTR than in those with the Ct value >37.5. Of the patients who received adjuvant chemotherapy, patients with mSEPT9 Ct value ≤37.5 had worse TTR than in those with the Ct value >37.5 (Figure 3C), which was consistent with the validation study result (Figure 3D). Moreover, the Ct value of mSEPT9 did not affect the TTR related to dMMR and KRAS/NRAS mutations, although the status of mSEPT9 (Ct value ≤37.5) reduced the TTR of patients with BRAF or PIK3CA mutations. This suggested that mSEPT9 could affect the mutations in members downstream to the EGFR signaling pathway and further affect the treatment outcome with adjuvant chemotherapy.

**TABLE 5:** Univariate and multivariate Cox proportional hazard analysis of cancer-specific survival (stages Ⅰ–Ⅳ) and time to recurrence (stages I–Ⅲ).

Variable	No. of patients	Cancer-specific survival (CSS)				Time to recurrence (TTR)			
		Univariate		Multivariate		Univariate		Multivariate	
		HR (95% CI)	*p*	HR (95% CI)	*p*	HR (95% CI)	*p*	HR (95% CI)	*p*
**mSEPT9 Ct value**									
>37.5	177	1 (ref)		1 (ref)		1 (ref)		1 (ref)	
≤37.5	120	2.52 (1.75–3.32)	0.003	2.01 (1.02–3.55)	0.005	2.27 (1.58–3.83)	0.004	1.98 (2.26–3.67)	0.009
**mSEPT9 status**									
Negative	31	1 (ref)		1 (ref)		1 (ref)		1 (ref)	
Positive	266	1.08 (0.64–1.69)	0.35	1.02 (0.54–1.60)	0.47	1.12 (0.67–1.72)	0.28	1.06 (0.61–1.63)	0.39
**TNM stage**									
Stage I	24	1 (ref)		1 (ref)		1 (ref)		1 (ref)	
Stage II	128	1.73 (1.20–2.49)	0.02	1.45 (0.98–2.82)	0.045	1.82 (1.41–2.66)	0.01	1.59 (0.98–2.22)	0.032
Stage III	120	3.08 (2.14–4.42)	0.0005	2.82 (1.91–3.93)	0.001	2.91 (2.02–4.15)	0.0008	2.65 (2.06–3.67)	0.002
Stage IV	25	5.90 (4.34–9.41)	0.0001	4.75 (3.16–8.29)	0.0003	5.05 (3.51–8.22)	0.0002	4.22 (2.67–7.38)	0.0004
**MMR status**									
dMMR	59	1 (ref)		1 (ref)		1 (ref)		1 (ref)	
dMMR + mSEPT9	32	0.91 (0.59–1.63)	0.42	0.97 (0.61–1.66)	0.50	0.95 (0.64–1.70)	0.47	0.99 (0.64–1.70)	0.58
pMMR	238	1.15 (0.49–1.71)	0.25	1.08 (0.67–1.69)	0.36	1.21 (0.82–1.99)	0.18	1.16 (0.70–1.75)	0.22
pMMR + mSEPT9	99	1.07 (0.60–1.63)	0.38	1.01 (0.54–1.58)	0.40	1.09 (0.69–1.73)	0.32	1.05 (0.61–1.63)	0.42
***KRAS*/*NRAS* status**									
Wild-type	151	1 (ref)		1 (ref)		1 (ref)		1 (ref)	
Wild + mSEPT9	72	1.09 (0.61–1.68)	0.35	1.03 (0.52–1.57)	0.40	1.13 (0.80–1.76)	0.26	1.02 (0.55–1.59)	0.43
Mutated	146	2.78 (1.93–3.63)	0.002	2.56 (1.73–3.42)	0.005	2.92 (2.01–3.98)	0.001	2.76 (1.89–3.77)	0.006
Mutated + mSEPT9	86	1.17 (0.72–1.78)	0.20	1.28 (0.85–1.86)	0.14	1.11 (0.69–1.75)	0.42	1.01 (0.57–1.60)	0.42
***BRAF* status**									
Wild-type	282	1 (ref)		1 (ref)		1 (ref)		1 (ref)	
Wild + mSEPT9	139	1.13 (0.63–1.72)	0.25	1.07 (0.55–1.68)	0.33	1.21 (0.74–1.83)	0.13	1.15 (0.68–1.79)	0.22
Mutated	15	2.67 (1.86–3.49)	0.002	2.46 (1.61–3.22)	0.004	2.72 (1.91–3.68)	0.001	2.69 (1.85–3.51)	0.002
Mutated + mSEPT9	13	2.62 (1.85–3.52)	0.002	2.38 (1.49–3.01)	0.005	2.69 (1.88–3.92)	0.002	2.51 (1.71–3.50)	0.003
***PIK3CA* status**									
Wild-type	291	1 (ref)		1 (ref)		1 (ref)		1 (ref)	
Wild + mSEPT9	128	0.96 (0.58–1.62)	0.55	0.98 (0.57–1.63)	0.62	1.05 (0.60–1.71)	0.30	1.02 (0.59–1.67)	0.37
Mutated	6	1.06 (0.66–1.75)	0.40	1.02 (0.58–1.65)	0.44	1.08 (0.65–1.77)	0.37	1.03 (0.69–1.74)	0.40
Mutated + mSEPT9	5	1.51 (1.02–2.29)	0.041	1.32 (0.79–2.01)	0.055	1.58 (1.10–2.28)	0.038	1.53 (1.06–2.33)	0.049

### The Prognostic Effect of Methylated Septin9 on Specific Genotype Mutation in Different Colorectal Cancer Stages

Patients with stage I–Ⅲ CRC with mSEPT9 Ct value ≤37.5 had significantly worse CSS than those with mSEPT9 Ct value >37.5. There was no difference in CSS in patients with stage Ⅳ CRC. Patients with stage I–II CRC with the dMMR status was independently associated with better CSS but not in those with stage Ⅲ and stage IV CRC. Moreover, dMMR with positive mSEPT9 (Ct value ≤37.5) in stage Ⅲ CRC was associated with worse CSS than that with dMMR. No differences in CSS were observed in patients with stage I–II CRC irrespective of mutated or wild-type status of KRAS/NRAS/BRAF/PIK3CA, although significant differences in CSS was observed for stage III and IV patients with mutated and wild-type tumors. Additionally, the BRAF- or PIK3CA-mutated tumors with positive mSEPT9 (Ct value ≤37.5) had shorter CSS in stages Ⅲ and Ⅳ than the tumors with Ct value of mSEPT9 > 37.5 (Table 6).

**TABLE 6:** Cox analysis of prognostic variables for CSS in colorectal patients from stage I to stage IV.

Variable	Stages I–II		Stage III		Stage IV	
	HR (95% CI)	*p*	HR (95% CI)	*p*	HR (95% CI)	*p*
**mSEPT9 Ct value**						
>37.5	1.57 (0.74–3.36)	0.28	1.42 (0.65–3.11)	0.44	1.21 (0.41–3.05)	0.66
≤37.5	2.26 (1.19–4.29)	0.01	2.17 (1.08–4.21)	0.04	2.01 (0.81–3.98)	0.13
dMMR	0.79 (0.71–0.92)	0.02	1.34 (0.55–3.09)	0.51	1.25 (0.44–3.10)	0.58
dMMR + mSEPT9	1.46 (0.62–3.13)	0.40	2.20 (1.10–4.22)	0.03	1.59 (0.75–3.41)	0.32
*KRAS*/*NRAS* mutated	1.98 (0.76–3.85)	0.07	2.41 (1.43–4.52)	0.01	4.08 (1.62–11.30)	0.001
*KRAS*/*NRAS* mutated + mSEPT9	1.93 (0.75–3.79)	0.12	1.67 (0.85–3.45)	0.25	1.72 (0.92–3.49)	0.21
*BRAF* mutated	1.89 (0.71–3.81)	0.16	2.22 (1.15–4.28)	0.04	2.53 (1.39–5.02)	0.005
*BRAF* mutated + mSEPT9	1.60 (0.74–3.39)	0.30	2.51 (1.35–4.87)	0.006	2.88 (1.49–8.12)	0.004
*PIK3CA* mutated	1.73 (0.59–3.64)	0.20	2.19 (1.08–3.18)	0.03	2.37 (1.38–4.71)	0.008
*PIK3CA* mutated + mSEPT9	1.81 (0.64–3.73)	0.18	2.72 (1.41–7.29)	0.004	3.12 (1.55–9.25)	0.002

### The Role of Methylated Septin9 in the Evaluation of Surgical Effectiveness in Stage Ⅰ–Ⅲ Colorectal Cancer

To study the prognostic value of mSEPT9 in a surgical effectiveness and TTR in patients with stage I–III CRC, we tested peripheral mSEPT9 levels 7 days after surgery and simultaneously collected clinical regular examination results for CEA. The recurrence rate in patients with positive mSEPT9 was higher than those with positive CEA. Furthermore, the recurrence rate in patients with mSEPT9 combined with CEA was higher than in those with mSEPT9 alone, which indicated that mSEPT9 was a useful predictive marker for the evaluation of postsurgical prognosis of CRC (Table 7; Figure 3E).

**TABLE 7:** Comparison of prognosis for TTR between mSEPT9 and CEA after radical operation.

	Positive number	Number of recurrence	Rate of recurrence (%)
mSEPT9	22	15	68.2
CEA	51	16	31.4
mSEPT9 + CEA	15	13	86.7

## Discussion

Early diagnosis and treatment is the key to improve prognosis of CRC patients. Lofton-Day C. et al. detected the expression of methylated *TMEFF2*, *NGFR,* and *SEPT9* genes in plasma samples of CRC patients and healthy controls using the reverse transcription polymerase chain reaction, and the results confirmed that the methylated *SEPT9* gene was highly expressed in cancer tissues and the peripheral blood of CRC patients (Lofton-Day et al., 2008). The high sensitivity and specificity of mSEPT9 in peripheral blood makes it an ideal tool for the screening of CRC, which can be carried out using liquid biopsy technology molecular indicators for circulating tumor DNA (ctDNA). At present, FOBT and endoscopy are the predominant methods for early screening of patients with a high risk of CRC. However, given the poor sensitivity and specificity of FOBT, screening of CRC using FOBT cannot effectively improve the compliance for endoscopy. Our previous experiments showed that mSEPT9 was superior to FOBT in the specificity and sensitivity for early screening of CRC (Sun et al., 2019). Here, we analyzed whether the application of mSEPT9 could improve the compliance of endoscopy detection, and the results showed that the rate of endoscopy examination in patients with positive mSEPT9 was significantly higher than those with positive FOBT.

Owing to the heterogeneity of the tumor reflected in multiple pathway-related molecular alterations, there is considerable stage independent variability in clinicopathological performance (Kim et al., 2013; Sinicrope et al., 2017; Guo et al., 2019). The DNA repair system regulates the pathogenesis of CRC, and the loss or reduction in function of dMMR cells to repair mismatched bases leads to the formation of MSI, instability of the MSI-related genome, and an increase in tumor susceptibility (Dienstmann et al., 2019). Most of the microsatellite sequences are located in the coding region of genes involved in oncogenesis and development. The absence of the MMR system can inhibit the tumor suppressor genes and activate oncogenes (Lochhead et al., 2013). The frequency of chromosomal aberrations in CRC patients is 50–85%. Chromosomal instability can lead to the activation of oncogenes, such as the Ras, and inactivation of tumor suppressor genes, such as APC and TP53, which usually reduce the duration of overall survival and progression free survival and cause adverse prognosis after treatment with 5-fluorouracil (Liu et al., 2015; Aghagolzadeh and Radpour, 2016). CIMP is closely related to many molecular features, including MSI, epigenetic silencing of mismatch repair gene *MLH1*, and mutations in *TP53*, *BRAF,* and *KRAS* (Dawson et al., 2014; Koelzer et al., 2015; Vedeld et al., 2017)*.* Moreover, CIMP is also associated with few clinicopathological features, including tumor localization, gender, age, tumor type, differentiation, and so on. A few studies have shown that left-sided colon cancer is mainly characterized by chromosome instability, abnormal activation of the growth factor signaling pathway and the Wnt signaling pathway, and well differentiation. Whereas, right-sided colon cancer is characterized by high microsatellite instability, high methylation of CpG island, mutations in key carcinogenic proteins, mutations in *BRAF*, and expression of oncoproteins in the serrated adenoma (Tejpar et al., 2017). The pathological phenotype of right-sided colon cancer is poor differentiation, high proportion of advanced disease, increased mucinous adenocarcinoma, and high propensity for complications and second primary intestinal tumor that associates with poor prognosis (Missiaglia et al., 2014; Sveen et al., 2019).

Further, a previous study observed differences in the positive rate of *SEPT9* methylation in the peripheral blood of CRC patients with varied clinicopathological characteristics, and its positive rate is significantly related to the malignant biological behavior (Shen et al., 2019). Our previous studies showed that the methylation of *SEPT9* is associated with the TNM stage, total tumor volume, and mismatch repair deficient status (Sun et al., 2019), suggesting that mSEPT9 in peripheral blood may be related with the pathological stage and the prognosis of patients. In this study, we examined mSEPT9, the status of TP53 and DNA mismatch repair, and mutations in KRAS, NRAS, BRAF, and PIK3CA and their association with clinicopathological characteristics. The results indicated that the level of mSEPT9 in the peripheral blood of CRC patients was associated with missense mutations in TP53. The mSEPT9 levels could induce loss of dMMR-specific gene expression, especially the MLH1 gene. Furthermore, the levels of mSEPT9 were related to the mutations in BRA*F* and *PIK3CA*, consistent with results of previous studies (Fu et al., 2012; Sun et al., 2014; Kang et al., 2015). Furthermore, the Ct values of mSEPT9 before surgery were found to be associated with the overall prognosis of CRC patients, and the prognosis of patients who received adjuvant chemotherapy. It might be of greater clinical significance to analyze the combined prognostic evaluation value of mSEPT9 and different molecular indicators. Results of our study showed that mSEPT9 aggravated the influence on the prognosis of CRC patients with dMMR of stage Ⅲ and BRAF/PIK3CA gene mutation of stages Ⅲ–Ⅳ. However, no correlation was observed between mSEPT9 and patients' gender, tumor location, and differentiation.

About 30% of patients with stage III CRCs are prone to recur despite the administration of adjuvant chemotherapy. It is necessary to use an effective strategy after surgery to evaluate its therapeutic effect and provide a timely assessment and adjustment of the therapy (Shi et al., 2013). Serum CEA is a common marker recommended for recurrence monitoring of CRC in the clinics. However, its sensitivity and specificity are suboptimal. Studies have shown that the levels of mSEPT9 in peripheral blood decrease or become negative after radical surgery of CRC and then again become positive for tumor recurrence (Song et al., 2018), which suggests that mSEPT9 may be a molecular marker for monitoring the recurrence and metastasis of CRC. Furthermore, we analyzed the relationship between the results of mSEPT9 and TTR 7 days after operation, which showed that patients with positive mSEPT9 were significantly associated with worse TTR than in those with negative mSEPT9. This indicated that mSEPT9 may be helpful to predict the effect and outcome of surgery. Moreover, the prediction of CRC residual lesions after surgery using mSEPT9 was better than that using CEA, and the combined detection using mSEPT9 and CEA could improve the accuracy of postoperative evaluation of CRC.

## Conclusion

The association of mSEPT9 with clinicopathological and molecular characteristics suggests that mSEPT9 may serve as an effective biomarker for screening and assessing the prognosis of CRC. Further study of mSEPT9 could optimize the molecular subtypes and improve the individualized treatment of CRC. In future, we will increase the sample size and extend the follow-up time to clarify the clinicopathological efficacy of mSEPT9 detection. Targeted therapy against hypermethylation of CpG island would play an important role in the treatment of CRC.

## Data Availability

The original contributions presented in the study are included in the article/Supplementary Material, and further inquiries can be directed to the corresponding author.

## Abbreviations

CEA, carcinoembryonic antigen; CIMP, CpG island methylator phenotype; CIN, chromosome instability; CRC, colorectal cancer; Ct, cycle threshold; dMMR, mismatch repair deficiency; FOBT, fecal occult blood test; mSEPT9, methylated Septin9; MSI, microsatellite instability; CSS, cancer-specific survival; TTR, time to recurrence; EGFR, epidermal growth factor receptor.
